# Editorial: Physiologic signals in neonatal intensive care

**DOI:** 10.3389/fped.2022.973189

**Published:** 2022-07-14

**Authors:** Eric W. Reynolds

**Affiliations:** Division of Neonatal-Perinatal Medicine, Department of Pediatrics, McGovern Medical School at the University of Texas Health Science Center at Houston, Houston, TX, United States

**Keywords:** physiologic signals, heart rate variability, vital sign analysis, feeding rhythmicity, delayed cord clamping

Physiological signals are generated by the body during both the normal and pathologic functioning of various physiological systems. Humans emit a wide range of physiologic signals that can be monitored by clinicians and used to diagnose illness, assess the effectiveness of treatments and predict short- and long-term outcomes. These signals can be electrical, mechanical, acoustic, magnetic, chemical, impedance or optical. Interpreting these signals is particularly important when the patients are non-communicative, a ubiquitous problem in the neonatal intensive care unit.

The first known report of palpating a pulse comes from the Epic of Gilgamesh, the oldest written story known to exist (1). Upon the death of his friend, Gilgamesh laments, “I touch his heart and it does not beat at all” and, with that, the first physiologic signals were documented (1). The first reported measurement of blood pressure occurred in 1733 when Sir Stephen Hales accessed the leg artery of a horse with a glass tube and reported the column of blood rose to “8 feet, 3 inches above the left ventricle” (2). The first mercury manometer was described in 1828 by Jean Leonard Marie Poiseuille (2). In 1856, Köllicker and Muller discovered that heart muscle produces electricity (3). A few years later, around 1870, Müirhead recorded the first electrocardiogram, which was improved in 1950 when it was paired with Elmqvist's direct-writing inkjet recorder (3). Similarly, electrical signals from brain activity were documented by Hans Berger in 1929 with his first report of the use of electroencephalography (4).

Advances in microchip technology, computer processor speed, and signal transducer miniaturization have improved our ability to access information produced by the body and collect extremely large datasets. Digital conversion and manipulation of these datasets combined with real-time analysis technologies are continually improving the clinician's ability to interpret these signals. Notable among these advances is the HeRO Score, which monitors both high and low frequency heart rate variability as an early sign of neonatal sepsis (5).

In this special issue of Frontiers in Neonatology, we have collected works from animal research, literature review and human clinical studies to focus a discussion of physiologic signals in the neonatal intensive care unit. Blank et al., describe their study of umbilical cord management at birth in sheep, showing that hypothermia is not a significant risk during delayed cord clamping. Patra et al., discuss a wide range of physiologic signals associated with a hemodynamically significant patent ductus arteriosus. Harvey-Jones et al., review the modalities to monitor, treat and predict outcomes of infants with neonatal encephalopathy. Salverda et al., describe how sample-rate presents a common problem for analysis of extremely large data sets. In their retrospective study of inspired oxygen and oxygen saturation levels, they found little difference between 1-per-second samples and 1-per-minute samples. While this observation can make the data sets more manageable, the relationship may not hold true for all types of physiologic signals in all settings. Javorka et al., assessed beat-to-beat blood pressure variability to describe the maturation of baroreflex sensitivity linked to postmenstrual age rather than postnatal age. Lee et al., demonstrated the feasibility of monitoring infant sleep-wake cycles using a non-contact method, impulse-radio ultrawideband radar, compared to other techniques (video, amplitude-integrated EEG and actigraphy). While not ready for clinical application, it does represent a continued advance in neonatal monitoring and a promising future for the field.

Finally, Gewolb et al., describe the effect of neurologic injury on the biorhythms of feeding in premature infants. Gewolb et al. succumbed to complications of liver failure in 2021. A major focus of his career was on the study of suck-swallow-breath rhythms during newborn feeding and how those physiologic signals in the newborn period may be used to predict long-term neurologic outcomes. His other publications in this area looked at the effects of prematurity, drug exposure and lung disease on suck-swallow-breath coordination. Over his career, he published nearly 100 original manuscripts. He mentored many neonatal fellows to their own successful academic and clinical careers. He was instrumental to the founding of the Chinese Journal of Contemporary Pediatrics and was honored for his contribution at the twentieth anniversary of the journal in 2019. His work contained in this issue of Frontiers, describing the increased variability in feeding biorhythms due to intraventricular hemorrhage will be one of his final publications before his death. He was a great teacher, mentor and friend. He will be missed.

As technology continues to improve in general, we can expect the field of physiologic signals to grow. Faster computers and smaller hardware will give clinicians easier access to more, large-volume data sets with real-time and automated analyses. These tools will help clinicians treat patients and improve outcomes.

## Author contributions

The author confirms being the sole contributor of this work and has approved it for publication.

## Conflict of interest

The author declares that the research was conducted in the absence of any commercial or financial relationships that could be construed as a potential conflict of interest.

## Publisher's note

All claims expressed in this article are solely those of the authors and do not necessarily represent those of their affiliated organizations, or those of the publisher, the editors and the reviewers. Any product that may be evaluated in this article, or claim that may be made by its manufacturer, is not guaranteed or endorsed by the publisher.
